# Data on wind-induced responses of the hanging point for a high-speed railway in China

**DOI:** 10.1016/j.dib.2018.11.080

**Published:** 2018-11-19

**Authors:** Qiang Xie, Xi Zhi

**Affiliations:** a College of Civil Engineering, Tongji University, No. 1239 Siping Road, Yangpu District, Shanghai, China; b Key Laboratory of Performance Evolution and Control for Engineering Structures (Tongji University), Ministry of Education, China

## Abstract

A recent study by Xie and colleagues show the dynamic behavior of the catenary system in turbulent and uniform flow conditions under four different tension combinations (Xie et al., 2018). The data presented in this article include the maximum displacement and maximum acceleration of the hanging point along the wind direction, comparing the values in two different wind flows and four different tension combinations. These data are useful to explore the influence and reveal the resonance interaction between structural elements and contact wire.

**Specifications table**

**Table:** 

Subject area	*Engineering*
More specific subject area	*Wind Engineering*
Type of data	*Text files*
How data was acquired	*A micro-acceleration sensor with high sensitivity and a laser displacement meter are used to measure the displacement (D*_*2×*_*) and acceleration (A*_*1×,y*_*) at the hanging point of the steady arm.*
Data format	*Raw data statistically analyzed*
Experimental factors	*A micro-acceleration sensor with the frequency of 1000 Hz and a laser displacement meter with the frequency of 500 Hz are used to collect data for 60 s for each test condition.*
Experimental features	*Wind tunnel tests of an aeroelastic model with five masts and four spans were carried out with the design requirements of a catenary system and similarity theory to research wind characteristics. The acceleration and displacement of the hanging point were measured using sensors.*
Data source location	*Tongji University, Shanghai city, China*
Data accessibility	*With this article*

**Value of the data**

• The data provide the maximum displacement and acceleration of the hanging point along the wind direction.
• The data allow a comparison under turbulent and uniform flows and four kinds of tension combinations on wires.
• The data are useful to explore the influence on displacement and acceleration of the hanging point.
• Further analysis of the data presented here may reveal the resonance interaction between structural elements and contact wire.

## 1 Data

Data reported here describe the maximum displacement (Table 1) and maximum acceleration (Table 2) of hanging point along the wind direction, comparing the values in two different wind flows and four different tension combinations [1]. The uniform flow and the turbulence flow with the intensity of 20% have been chosen. Tension combinations are classified into four grades by the rail speed. The tensions in the contact wire are 147 N (Grade 1), 137 N (Grade 2), 118 N (Grade 3) and 98 N (Grade 4). In Table 1, the values for Grade 2 in uniform flow are missing due to the sensor dropping during the experiment.

**Table 1:** Maximum displacement of hanging point along the wind direction.

Flow	Max displacements (mm)	Wind velocities (m/s)								
		3	4	5	6	7	8	10	12	14
Uniform flow	Grade 1	0.02	0.04	0.06	0.08	0.12	0.16	0.25	0.40	0.55
	Grade 2	0.03	0.04	0.07	0.09	0.12	0.16	0.26	0.41	0.56
	Grade 3	0.02	0.03	0.05	0.06	0.09	0.14	0.26	0.40	0.60
	Grade 4	0.03	0.05	0.07	0.10	0.13	0.18	0.30	0.45	0.61
Turbulence flow	Grade 1	0.03	0.05	0.07	0.12	0.18	0.28			
	Grade 2									
	Grade 3	0.03	0.06	0.09	0.14	0.21	0.29			
	Grade 4	0.04	0.07	0.10	0.15	0.22	0.32

**Table 2:** Maximum acceleration of hanging point along the wind direction.

Flow	Max displacements (mm)	Wind velocities (m/s)								
		3	4	5	6	7	8	10	12	14
Uniform flow	Grade 1	0.34	0.60	0.65	0.98	1.40	1.59	2.63	4.53	7.03
	Grade 2	0.48	0.54	0.80	1.04	1.46	1.48	2.61	4.64	7.21
	Grade 3	0.58	0.54	0.73	0.98	1.50	1.92	2.83	4.71	7.33
	Grade 4	0.58	0.71	0.91	1.08	1.44	1.61	2.60	4.58	7.96
Turbulence flow	Grade 1	0.63	0.94	1.37	1.83	2.59	3.88			
	Grade 2	0.76	1.08	1.60	1.85	2.93	4.79			
	Grade 3	1.35	1.49	2.00	2.36	2.75	4.04			
	Grade 4	0.69	1.07	1.56	2.37	2.90	4.21			

## 2 Experimental design, materials and methods

The prototype of this study is part of the catenary system. The model catenary system was designed based on similarity theory [2], [3]. Limited by the height of wind tunnel, the geometric similarity ratio of structure elements λ_L_ was restricted to 1:4.5, and making the acceleration similarity ratio *λ*_a_ being 1:1. A micro-acceleration sensor with high sensitivity and a laser displacement meter are used to measure the displacement (*D*_2×_) and acceleration (*A*_1×,y_) at the hanging point of the steady arm. The displacement data *D*_2×_ are collected for 60 s by the laser displacement meter with the frequency of 500 Hz. Micro acceleration sensor with the frequency of 1000 Hz is used to collect the acceleration data *A*_1×,y_ for 60 s.
