# Supplementary material for: Data on wind-induced responses of the hanging point for a high-speed railway in China
Source: Data Brief. 2018 Nov 19;21:2259–61. doi: 10.1016/j.dib.2018.11.080 (PMC6276545; doi:10.1016/j.dib.2018.11.080)
Supplement: Supplementary file 1 — Supplementary material [file mmc1.docx]

**Conflict of interest**

We wish to confirm that there are no known conflicts of interest associated with this publication. (Author: Qiang Xie, Xi Zhi）
